# AT1 Receptor Blockade Attenuates Insulin Resistance and Myocardial Remodeling in Rats with Diet-Induced Obesity

**DOI:** 10.1371/journal.pone.0086447

**Published:** 2014-01-23

**Authors:** Silvio A. Oliveira-Junior, Paula F. Martinez, Danielle M. Guizoni, Dijon H. S. Campos, Tiago Fernandes, Edilamar M. Oliveira, Marina P. Okoshi, Katashi Okoshi, Carlos R. Padovani, Antonio C. Cicogna

**Affiliations:** 1 Botucatu Medical School, São Paulo State University, Botucatu, Brazil; 2 School of Physiotherapy, Federal University of Mato Grosso do Sul, Campo Grande, Brazil; 3 School of Physical Education and Sport, University of São Paulo, São Paulo, Brazil; 4 Botucatu Biosciences Institute, São Paulo State University, Botucatu, Brazil; Max-Delbrück Center for Molecular Medicine (MDC), Germany

## Abstract

**Background:**

Although obesity has been associated with metabolic and cardiac disturbances, the carrier mechanisms for these responses are poorly understood. This study analyzed whether angiotensin II blockade attenuates metabolic and cardiovascular disorders in rats with diet-induced obesity.

**Material and Methods:**

Wistar-Kyoto (n = 40) rats were subjected to control (C; 3.2 kcal/g) and hypercaloric diets (OB; 4.6 kcal/g) for 30 weeks. Subsequently, rats were distributed to four groups: C, CL, OB, and OBL. L groups received Losartan (30 mg/kg/day) for five weeks. After this period we performed in vivo glucose tolerance and insulin tolerance tests, and measured triacylglycerol, insulin, angiotensin-converting enzyme activity (ACE), and leptin levels. Cardiovascular analyzes included systolic blood pressure (SBP), echocardiography, myocardial morphometric study, myosin heavy chain composition, and measurements of myocardial protein levels of angiotensin, extracellular signal-regulated (ERK1/2), c-Jun amino-terminal kinases (JNK), insulin receptor subunit *β* (*β*IR), and phosphatidylinositol 3-kinase (PI3K) by Western Blot.

**Results:**

Glucose metabolism, insulin, lipid, and ACE activity disorders observed with obesity were minimized by Losartan. Moreover, obesity was associated with increased SBP, myocardial hypertrophy, interstitial fibrosis and improved systolic performance; these effects were also minimized with Losartan. On a molecular level, OB exhibited higher ERK, *Tyr*-phosphorylated βIR, and PI3K expression, and reduced myocardial angiotensin and JNK expression. ERK and JNK expression were regulated in the presence of Losartan, while angiotensin, *Tyr*-*β*RI, total and *Tyr*-phosphorylated PI3K expression were elevated in the OBL group.

**Conclusion:**

Angiotensin II blockade with Losartan attenuates obesity-induced metabolic and cardiovascular changes.

## Introduction

Cardiac remodeling consists of many adaptive alterations to the heart aimed at maintaining myocardial performance in response to stress conditions, including mechanical and volumetric overload [1]. This process is particularly common in obesity [2] and is characterized by time-dependent evolution involving several adaptive changes which are clinically represented by changes in cardiac shape, size, and function [1], [3]. Experimental evidence has shown that diet-induced obesity is associated with cardiac remodeling, substantiated by contractile disturbances [4]–[8], hypertrophy [4]–[9], interstitial fibrosis [5], [7], and molecular expression changes in contractile proteins including myosin heavy chain (MyHC) β isoform synthesis [5], [7], and endocrine disturbances such as insulin and glucose metabolism disorders [6]–[9].

Several molecular signaling pathways can mediate cardiac remodeling in obesity, including parietal deformation, cytokines, and growth factors [10]. As a result, differential mechanisms can interact in multiple points of the cytosol supporting the development of molecular *cross-talk* during the cardiac remodeling progress [10]. Recently, several studies have shown important forms of molecular interaction between the signal transmission pathways that mediate insulin and angiotensin II (ANG II) actions in the heart [11]–[14]. Insulin actions are highly regulated by many factors, including a large number of endocrine, inflammatory, neural, and cell-intrinsic agents, which have been shown to be dysregulated in obesity [14]. In general, regulatory mechanisms attenuate metabolic signaling from insulin stimuli by decreasing tyrosine-phosphorylation of proteins members from insulin pathway, including insulin receptor β subunit (Rβ) and phosphatidylinositol 3-kinase (PI3K) messenger [15]. Consequently, insulin resistance is a common pathological state in which target cells fail to respond to normal stimuli from circulating insulin [15]–[17]. Obesity-associated insulin resistance is a major risk factor for type 2 diabetes and cardiovascular disease [17].

ANG II actions are initiated through interaction with G-protein coupled receptors, especially, the ANG II type 1 receptor (AT1R) [14]. As well as coupling with the heterotrimeric G proteins, tyrosine kinase activation is intimately involved in AT1R receptor signaling [10], [14]. Both non-receptor type tyrosine kinases [Src, Fyn, Yes, Pyk2, focal adhesion kinase, and Janus kinase 2] and receptor-type tyrosine kinases (epidermal growth factor and platelet-derived growth factor receptors) are activated by AT1R [11], [14]. These tyrosine kinases regulate downstream signaling mechanisms, including mitogen-activated protein kinase (MAPK) cascades, with activation of MAPK subtypes *extracellular signal-regulated kinase* (ERK) and *c-Jun N-terminal kinase* (JNK), thereby playing a critical role in ANG II cardiac remodeling actions [18]. Parallel to its effects on cardiac muscle, AT1R activation also affects metabolic actions from insulin, such as protein degradation and/or inhibition of tyrosine-phosphorylation of Rβ, insulin receptor substrate (IRS), and PI3K messenger [14], [16]. Therefore, activation states of the renin-angiotensin system contribute to AT1R stimulation and may support the occurrence of remodeling and insulin resistance in the heart [17].

As obesity is a metabolic condition characterized by chronic activation of the renin-angiotensin system [19]–[25], in this study we evaluated whether MAPK cascade activation by AT1R stimulation is associated with myocardial remodeling and has an inhibitory effect on tyrosine phosphorylation of insulin cascade proteins in hearts of obese rats. Although this possible cross-talk has not been clarified in studies with experimental diet-induced obesity models, our initial hypothesis is that obesity is associated with AT1R and MAPK activation and, as consequence, cardiac remodeling and insulin cascade inhibition due to tyrosine phosphorylation reduction of key insulin pathway messengers in the myocardium.

## Materials and Methods

### Experimental design, metabolic and biometry characterization

The experimental protocol was approved by the Ethics Committee on Experiments of Botucatu Medical School, Sao Paulo State University, in accordance with US National Institutes of Health “Guide for the Care and Use of Laboratory Animals” (NIH Publication No. 85–23, revised 1996) [26].

Male 60-day-old *Wistar-Kyoto* rats (n = 40) were randomly distributed into two groups: Control (C) and Obese (OB). Group C received commercial rat chow (RC Focus 1765) and OB received a palatable hypercaloric diet [4] for 30 weeks. After this period, animals were assigned to four groups: Control (C); Control with Losartan intervention (CL), Obese (OB), and Obese with Losartan intervention (OBL). In addition to their feeding program, CL and OBL groups received 30 mg/kg/day Losartan in their drinking water for an additional five weeks. Age matched animals from C and OB groups continued to receive respective dietary support for the corresponding period. Animals were individually housed under controlled conditions of 22–24°C, 50–70%RH, and time-controlled 12-hour light/dark cycles. All animals had free access to water and chow (50 g/d). Food consumption was measured daily and water intake and body weight (BW) were evaluated once a week. Weekly calorie intake was calculated: average weekly food consumption × diet energy density. Feed efficiency - the ability to convert calorie intake into BW - was determined by: mean BW gain (g)/total calorie intake (Kcal).

The hypercaloric diet model consisted of five different palatable diets (HD1, HD2, HD3, HD4, and HD5) which were prepared from a mixture of industrialized products and supplementary ingredients added to commercial rat chow. These diets were alternately administered, with each chow type offered for seven days. Dietary composition was analyzed by the Animal Nutrition and Improvement Laboratory, School of Agricultural Sciences, UNESP and was as detailed in a previous study [4].

Hypercaloric diets were isocaloric with ∼30% more energetic content than the standard commercial rat chow (4.6 Kcal/g vs 3.2 Kcal/g); HD2 and HD4 were richer in lipids only, HD1 and HD5 also had an important carbohydrate content, especially sucrose. Although HD3 had similar energetic density (4.5 Kcal/g) to the other hypercaloric diets, its composition was mainly based on surplus sucrose from water solution. This diet model is similar to many other interventions used in studies on diet-induced obesity [4]–[8].

After the 35 week experimental period, GTT and ITT were realized in all animals.

To make the GTT, animals were maintained in 12–15h fasting. Subsequently, a blood sample was collected from the tip of the tail in a heparinized tube. Blood glycemia (basal condition) level for each animal was immediately determined using a handheld glucometer (Accuchek Advantage; Roche Diagnostics Co., Indianapolis, IN). Then, an injection of 2 g/kg glucose (Sigma- Aldrich1, St Louis, MO, USA) was given intraperitoneally. Blood glycemia levels were measured after 30, 60, 90, 120, 180, and 240 min [8].

For ITT, rats were fasted for 6 h before submission to insulin tolerance test. After determination of basal glycemia condition, 1.5 IU/kg insulin (Novolin® R, Novo Nordisk, Bagsvaerd, Denmark) was infused intraperitoneally and glucose measured 5, 10, 15, 20, 25, and 30 min thereafter [27].

After 12-15h fasting, animals were anesthetized with sodium pentobarbital (50 mg/kg) and euthanized by decapitation. Animals were thoracotomized and adipose depots (AD) from visceral, retroperitoneal, and epidydimal sites were measured. The adiposity index (%) was obtained from the sum of the weights of individual fat pads: ΣAD×100/BW [28].

Trunk blood was collected and centrifuged at 3000 g for 15 min at 4°C. Serum was separated by centrifugation at 3000 g for 15 minutes at 48°C and stored at −80°C until further analysis. Serum fractions of glucose, cholesterol, and triacylglycerol were measured with an automatic enzymatic analyzer system (Technicon, RA-XT™ System, Global Medical Instrumentation, Minnesota, USA) and enzyme kits (Kovalent Diagnosis, Rio de Janeiro/RJ, Brazil). Serum concentrations of non-esterified fatty acid (NEFA) were determined as per Johnson and Peters [29] using colorimetric kits (WAKO, Wako Pure Chemical Industries, Osaka, Japan). Serum leptin and insulin concentrations were determined by ELISA using commercial kits (Linco Research Inc., St. Louis, MO, USA).

The insulin metabolism was studied using the homeostatic model assessment index (HOMA-IR) calculated by the following formula: HOMA-IR =  ¼[fasting GL (mmol/l) fasting insulin (mU/ml)]/22.5 [30]. All rats ate normally and regained their body weight within 1 d after this regimen. ACE activity in rat serum and tissue extracts were determined using Abz-FRK(Dnp)P-OH derivatives as substrates by continuously measuring fluorescence according to previous studies [31], [32].

### Cardiovascular study

At the end of the experiment (35 weeks), systolic blood pressure (SBP) was measured by the non-invasive tail-cuff method with a Narco BioSystems® Electro-Sphygmomanometer (International Biomedical, Austin, TX, USA) [33]. The average of two readings was recorded for each animal. To analyze in vivo heart structure and performance, all animals were weighed and evaluated via transthoracic echocardiographic examination performed with a commercially available echocardiography machine (Sonos 5500, Philips, Andover, MA, USA) equipped with a 12-MHz phased array transducer. All measurements were obtained by the same observer using the method recommended by the American Society of Echocardiography [34].

For morphometric analysis, myocardial samples were fixed with a 10% formol solution for 48 h and then embedded in paraffin blocks. Histological sections (7 µm) stained with hematoxylin-eosin were used to measure cardiomyocyte cross-sectional area (CSA); determined for at least 100 myocytes per slide [35]. Major and minor nuclear diameters were also measured to determination nuclear volume [36]; fifty nuclei from each animal were measured. Nuclear volume (V) was estimated using the formula for a prolate ellipsoid: V = πAB^2^/6; where A is the major diameter and B the minor diameter [36].

Collagen interstitial fraction was determined by Picrosirius red staining of myocardium sections, analyzed under polarized light. Histological images were obtained using a LEICA DM LS microscope at 40× magnification coupled to a computer equipped with *Image Pro-plus*, an image analysis program (Media Cybernetics, Silver Spring, Maryland, USA).

MyHC isoform analysis was performed in duplicate by sodium dodecyl sulfate polyacrylamide gel electrophoresis. Sample preparation methods and electrophoresis conditions are detailed in a previous study [4]. Myocardial tissue protein levels were analyzed by Western blot. Sample preparation methods and electrophoresis conditions are detailed in Martinez et al. [37]. Western blot analysis was performed using antibodies against angiotensin (sc-7419), ERK (sc-93), JNK (sc-137019), β subunit of insulin receptor (Rβ; sc-711), Tyr^1162/1163^ phosphorylated Rβ (sc-25103), PI 3-kinase subunit p85α (sc-1637), and Tyr^508^ phosphorylated PI 3-kinase p85α (sc-12929), purchased from Santa Cruz Biotechnology, Inc. (Santa Cruz, CA). Protein levels were normalized to those of GAPDH (6C5, sc-32233, Santa Cruz Biotechnology).

### Statistical analysis

Nutritional, metabolic, and cardiovascular variables were evaluated by two-way ANOVA. When significant differences were found (p<0.05), the *post hoc* Tukey's multiple comparisons test for parametric, or Dunn's test for non-parametric distributions was performed. Level of significance was considered to be 5%.

## Results

**Figure 1:**
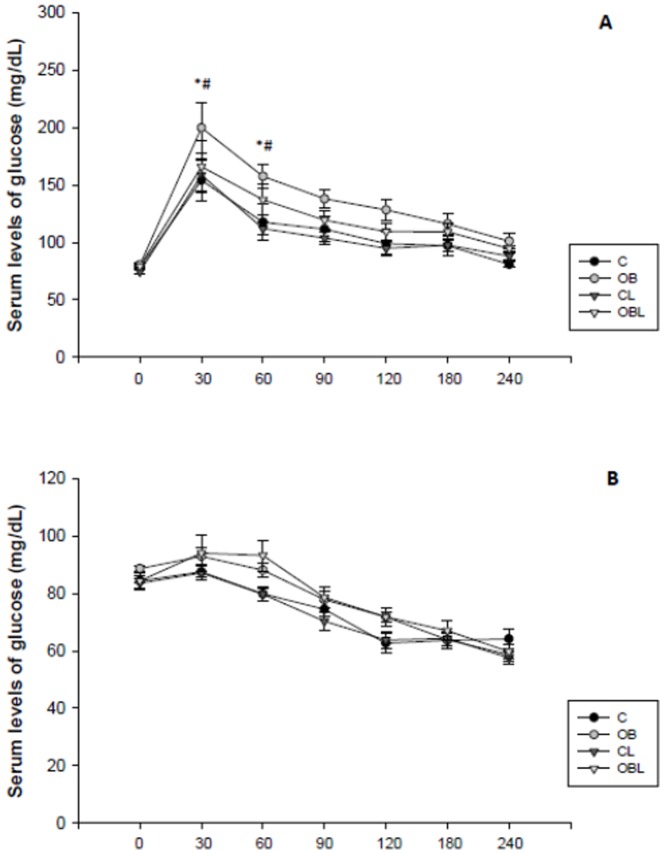
Serum glycemic levels following intraperitoneal glucose loads in (A) glucose tolerance test (GTT) and (B) insulin tolerance test; Data are expressed as means±SE; C: control group; CL: control group treated with losartan; OB: obese group; OBL: obese group treated with losartan; * p<0.05, OB vs. C group; #p<0.05, OB vs. OBL group; Repeated measures ANOVA and Bonferroni's test.

Although nutritionally, obesity has been associated with low calorie intake, OB groups exhibited higher feed efficiency, BW, and adiposity values compared to their respective Controls (p<0.01). Moreover, obesity *per se*, was also accompanied by elevated cholesterol, insulin, and insulin resistance levels, and leptinaemia. Also, obesity was characterized by higher triacylglycerol, NEFA and ACE activity leptinaemia as well as impaired glycemic tolerance, as seen by larger glycemic response AUC's in OB (Table 1) and by increased GTT glucose levels (Figure 1A).

**Table 1:** Nutritional and endocrine evaluation.

	Groups				Factors (p-value)		
Variable	C	OB	CL	OBL	Condition	Medication	Interaction
**Calorie intake(Kcal)**	**84.0±3.5**	67.3±5.7**	**84.2±5.2**	**68.6±8.7††**	**<0.001**	0.687	0.772
**Feed efficiency(mg/Kcal)**	**11.3±1.7**	19.6±2.9**	**11.3±1.8**	**19.1±3.3††**	**<0.001**	0.783	0.718
**Body weight(g)**	**557±44**	645±45**	**549±44**	**637±56††**	**<0.001**	0.597	0.988
**Adiposity(%)**	**5.9±1.0**	12.0±2.3**	**6.4±1.3**	**11.3±2.3††**	**<0.001**	0.946	0.321
**Cholesterol(mg/dl)**	**100.1±21.1**	122.2±7.7**	113.3±10.9*	125.6**±**12.1†	**<0.001**	0.065	0.267
**Triacylglycerol(mg/dl)**	**95±28**	122±35*	**96±19**	**102±17**	**0.050**	0.278	0.211
**NEFA(ng/dl)**	**0.32±0.03**	0.44±0.08**	0.41±0.04**	**0.39±0.07#**	**0.004**	0.354	<0.001
**Glucose(mg/dl)**	**126.8±21.3**	**139.9±15.9**	**145.3±22.6**	102.3**±**16.6†**#**	**0.128**	0.020	<0.001
**GTT(AUC)**	**26345±7530**	34841±5073**	**26519±3033**	**31300±6759**	**0.019**	0.319	0.387
**ITT(AUC)**	**2184±165**	**2341±144**	**2182±138**	**2383±292†**	**0.017**	0.947	0.570
**Insulin(ng/dL)**	**1.64±1.24**	2.89±1.17**	1.03±0.67*	**2.06±0.62†#**	**<0.001**	0.024	0.722
**HOMA-IR**	**10.2±8.4**	18.8±8.4**	**6.4±4.4**	**13.9±4.4†**	**<0.001**	0.046	0.803
**Leptin(ng/dL)**	**5.17±1.10**	9.71±1.85**	**5.29±1.25**	**10.27±2.38††**	**<0.001**	0.539	0.683
**ACE activity (UF·min^−1^·mg^−1^×1000)**	**90746±8544**	101126±9162*	104094±13117*	**94490±10252†**	**0.917**	0.370	0.011

Values expressed as mean ± SD; NEFA: non-esterified fatty acid; AUC: area under curve from glucose tolerance test response; HOMA-IR: homeostatic model assessment index; * p<0.05, ** p<0.01 vs. Control (C) group; #p<0.05, ##p<0.01 vs. OB; † p<0.05, †† p<0.01 vs. CL; ANOVA and Tukey test.

Although C and OB showed similar responses in ITT (Figure 1B), obesity was independently associated with higher AUC responses (p = 0.03; Table 1). With the exception of enzymatic activity and ITT, CL and OBL groups did not present differences in relation to metabolic and hormonal variables. Specifically, NEFA and ACE activity sustained obesity and Losartan interaction (p<0.05) while OB animals exhibited higher NEFA and ACE glycemic responses than C; Losartan attenuated these effects in OBL. Losartan independently reduced insulin values and HOMA-IR at both comparison levels (CL versus C and OBL versus OB; Table 1).

Taking into account *in-vivo* cardiovascular parameters, diet-induced obesity has been associated with increased systolic blood pressure, as seen between the non-medicated groups (p<0.05). Also, echocardiographic analysis showed lower left ventricular end-systolic diameter (LVESd) values, accompanied by higher interventricular septum diastolic thickness (IVST), posterior wall diastolic thickness (PWT), and left ventricle relative thickness in OB compared to C. Importantly, SBP, heart rate, IVST, and PWT values were lower with the medication, although the morphological differences in IVST and PWT were maintained between CL and OBL. Left ventricle relative thickness was similar between L groups (Table 2).

**Table 2:** Blood pressure and echocardiography study.

	Groups				Factors(p-value)		
Variable	C	OB	CL	OBL	Condition	Medication	Interaction
**SBP(mmHg)**	115±4	124±10*	109±8*	**112±14**	**0.050**	0.005	0.410
**Heart rate(beats/min)**	262±61	270±51	312±57*	306±42	**0.925**	0.015	0.672
**LA(mm)**	5.51±0.67	5.54±0.24	5.42±0.88	5.29±0.34	**0.790**	0.368	0.670
**LVEDd(mm)**	**8.43±0.71**	**8.20±0.40**	8.47±0.42	8.35±0.46	**0.288**	0.569	0.717
**LVESd(mm)**	**4.58±0.46**	4.10±0.48*	4.57±0.39	4.38±0.36	**0.017**	0.300	0.283
**PWT(mm)**	**1.51±0.04**	1.58±0.09*	1.46±0.06	**1.5±0.08†**	**0.003**	0.039	1.000
**IVST(mm)**	**1.53±0.04**	1.59±0.08*	1.47±0.06*	1.54±0.08#†	**0.007**	0.009	0.854
**LV relative thickness**	**0.36±0.03**	0.39±0.02*	0.35±0.02	**0.37±0.03**	**0.010**	0.071	0.857
**EFS(%)**	**45.8±2.3**	50.1±4.0*	**46.0±4.1**	**47.4±4.2**	**0.018**	0.300	0.223
**Ejection Fraction(ml)**	**0.84±0.02**	0.87±0.03*	**0.84±0.03**	**0.85±0.03**	**0.022**	0.281	0.268
**PWSV(mm/s)**	**34.1±3.0**	38.4±3.9*	**33.6±2.8**	**37.9±4.3†**	**0.002**	0.703	0.991
**E/A**	**1.62±0.32**	**1.58±0.16**	1.18±0.41*	**1.39±0.32**	**0.408**	0.004	0.213
**DTE(ms)**	**49.5±4.2**	**50.7±8.5**	**55.5±2.1**	**48.4±8.1**	**0.378**	0.581	0.223
**IVRT(ms)**	36.3±4.1	31.2±3.5**	31.5±4.1*	**28.5**±**2.6** #	**0.002**	0.004	0.391
**IVRT/R-R^0.5^**	**75.3±12.9**	65.8±8.7*	73.6±7.6	64.0±4.0†	**0.004**	0.577	0.985

Values expressed as mean ± standard deviation; SBP: systolic blood pressure; LA: left atrium diameter; LVEDd: left ventricular end-diastolic diameter; LVESd: left ventricular end-systolic diameter; PWT: posterior wall diastolic thickness of the left ventricle; IVST: interventricular septum diastolic thickness; LV relative thickness: relation between LV posterior wall systolic thickness and LVEDd; EFS: endocardium fraction shortening; PWSV: posterior wall shortening velocity; E/A: ratio between E and A waves evaluated in transmitral flow; DTE: E wave deceleration time; IVRT: LV isovolumetric relaxation time; IVRT/R-R0.5: ratio between IVRT and R-R heart rate interval; * p<0.05, ** p<0.01 vs. Control (C) group; #p<0.05, ##p<0.01 vs. OB; † p<0.05, †† p<0.01 vs. CL; ANOVA and Tukey test.

With respect to heart performance, endocardial fractional shortening (EFS), ejection fraction, and posterior wall shortening velocity (PWSV) were higher in OB than C. With the exception of PWSV, these alterations were attenuated by the Losartan, as CL and OBL presented similar systolic function results. In regard to diastolic performance, obesity reduced left ventricle isovolumetric relaxation time (IVRT) in absolute values and in relation to heart rate R-R interval (IVRT/R-R^0.5^). Losartan treatment reduced IVRT and E/A indexes in both L groups, maintaining similar results between CL and OBL groups (Table 2).

**Figure 2:**
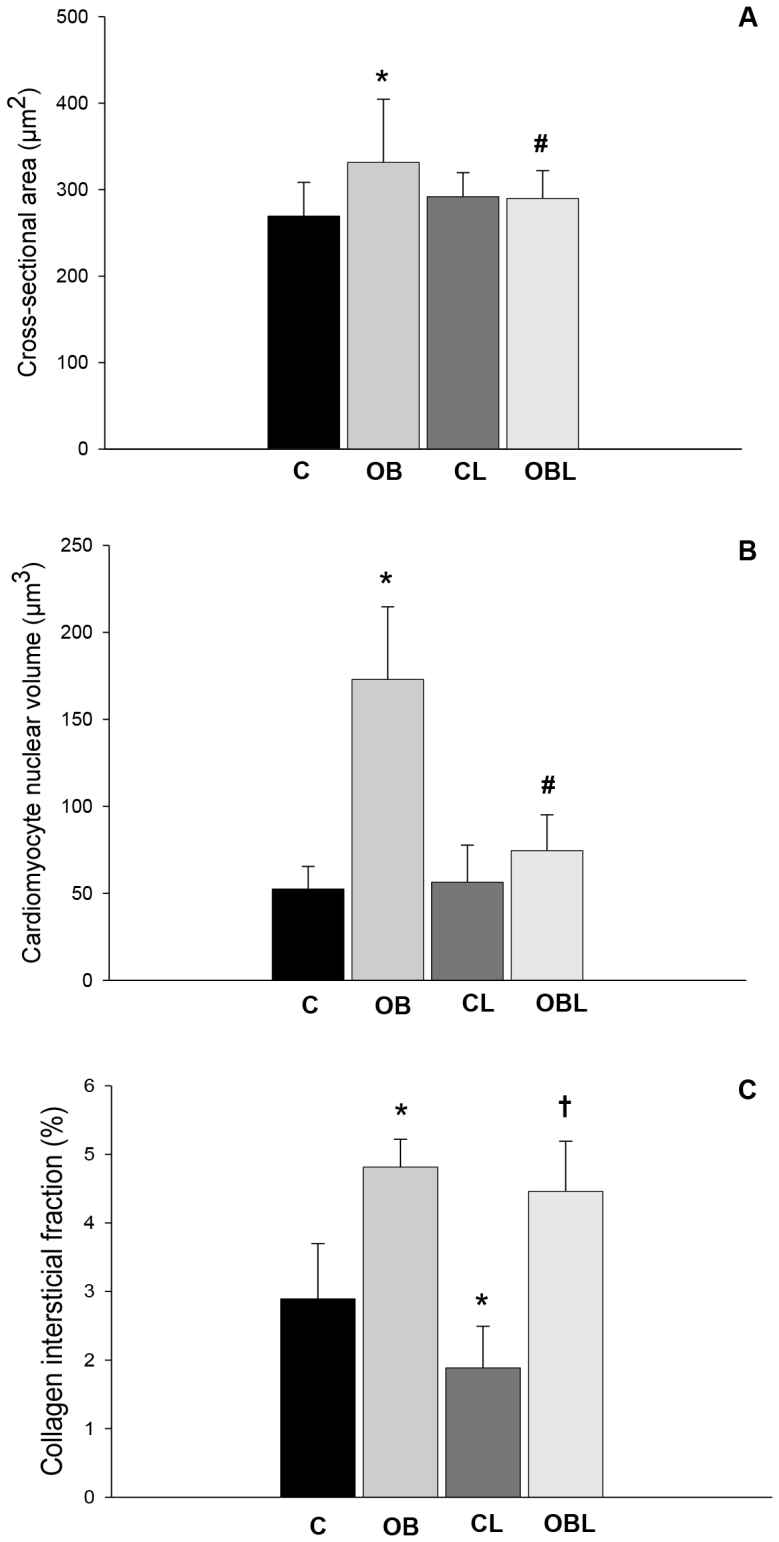
Morphometric analysis of the myocardial tissue; values presented as mean ± standard deviation. (A) Cardiomyocite cross-sectional area (µm^2^); (B) cardiomyocyte nuclear volume (µm^3^); (C) collagen interstitial fraction (%). C: control group; CL: control group treated with losartan; OB: obese group; OBL: obese group treated with losartan; * p<0.05 vs. C group; #p<0.05 vs. OB group; † p<0.05 vs. CL group; ANOVA and Tukey's test.

*Post-mortem* exam revealed a significant interaction between the condition and medication with respect to cardiomyocyte cross-sectional area (p = 0.034) and nuclear volume (p<0.001). Obesity increased cardiomyocyte cross-sectional area (C 269±12; OB 332±23; CL 292±9; OBL 290±10 µm^2^) and nuclear volume (C 6.56±0.54; OB 21.61±1.65; CL 7.26±0.70; OBL 9.32±0.81 µm^3^), demonstrated by OB compared to C (Figure 2A and 2B). Losartan administration reduced cross-sectional area and nuclear volume in OBL compared to OB.

**Figure 3:**
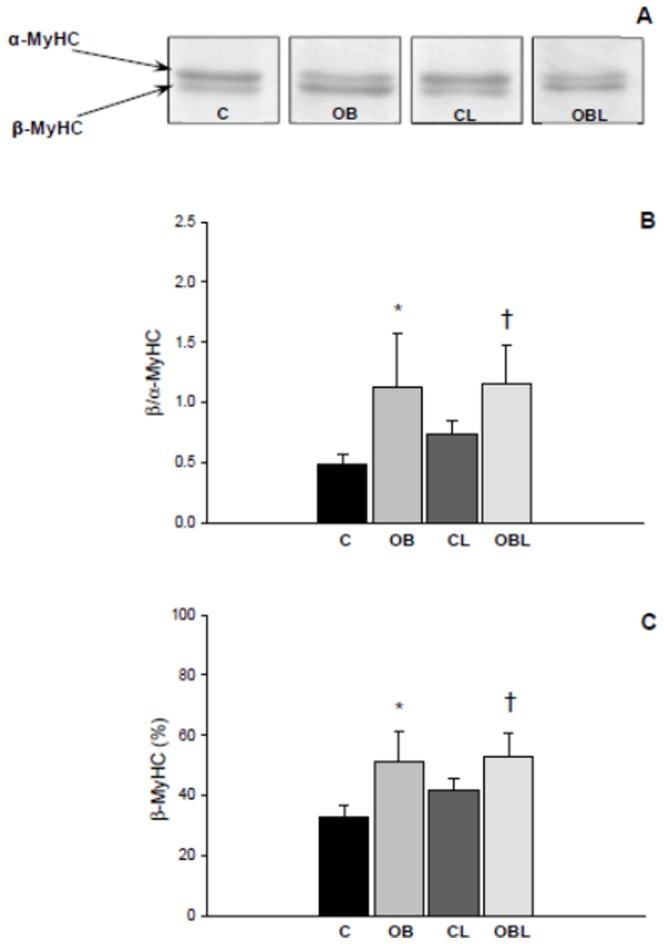
Myosin heavy chain (MyHC) expression in myocardial tissue; values presented as mean ± standard deviation. (A) β- and α-MyHC isoforms of the left ventricle; (B) relative contents (%) of β-MyHC; (C) ratio obtained from relationship between β- and α- MyHC expression. Electrophoresis conditions: sodium duodecyl sulfate polyacrylamide gel electrophoresis to 8%; running: 70 V, 20°C, 30-36 hours; sample concentrations: 10 µg/µl. Products were visualized with Coomassie brilliant blue staining. Band quantification was obtained by product densitometry analysis as integrated optical density. C: control group; CL: control group treated with losartan; OB: obese group; OBL: obese group treated with losartan; * p<0.05 vs. C group; #p<0.05 vs. OB group; † p<0.05 vs. CL group; ANOVA and Tukey's test.

In relation to interstitial collagen volume fraction (C 2.89±0.81; OB 4.81±0.41; CL 1.89±0.61; OBL 4.46±0.73%), obesity, as an independent factor, promoted interstitial fibrosis (p<0.001), while losartan, independently, attenuated collagen deposition in both medicated groups (p = 0.002; Figure 2C). Also, β/α MyHC ratio (C 0.49±0.08; OB 1.12±0.45; CL 0.73±0.12; OBL 1.16±0.32) and β MyHC content (C 33.0±3.5; OB 51.1±9.8; CL 41.8±3.8; OBL 52.8±7.9%) were higher in both obese groups (p<0.001; Figure 3A, B, and C). Also, AT1R blockade on its own reduced interstitial collagen fraction, but did not change β MHC content in the L groups.

**Figure 4:**
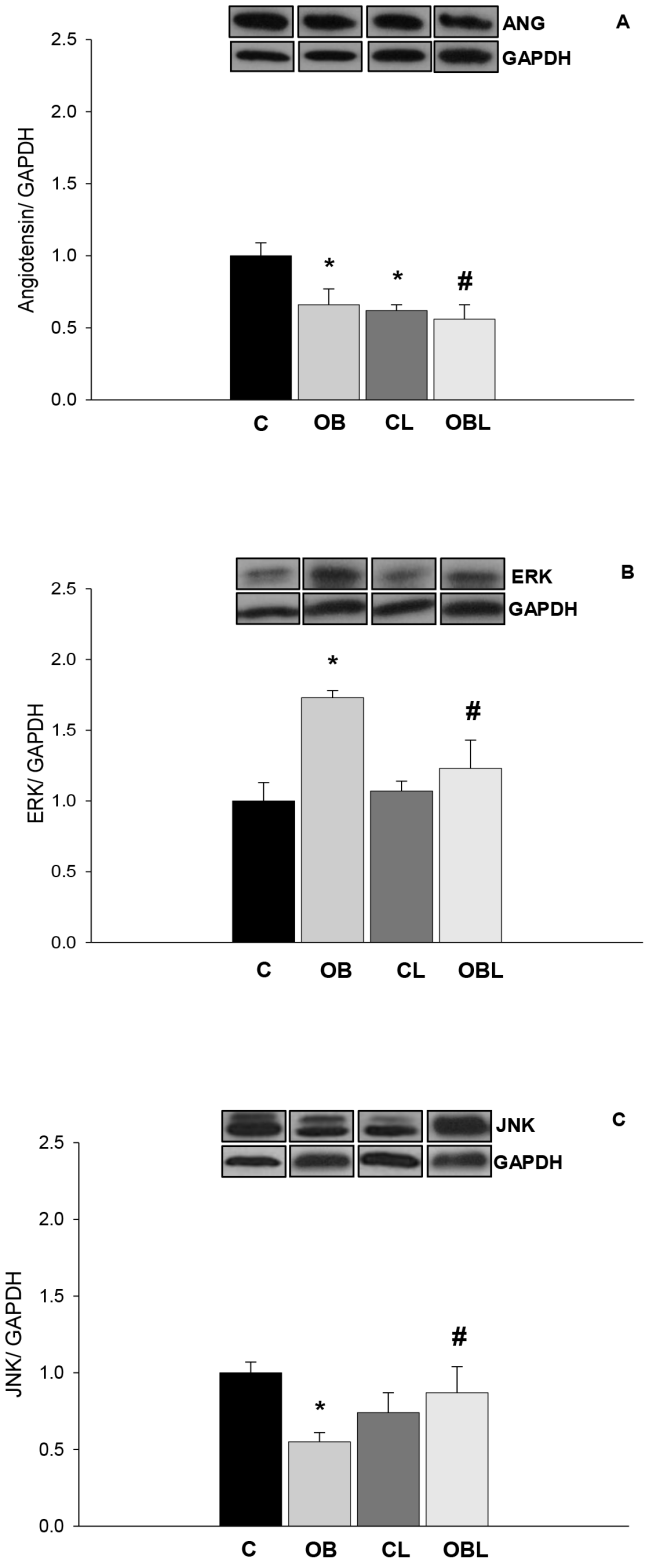
Protein levels of angiotensin and MAPK isoforms in myocardial tissue; values presented as mean ± standard deviation. Protein levels were normalized to GAPDH levels. (A) Angiotensin levels; (B) ERK levels; (C) JNK levels; C (n = 6): control group; CL (n = 6): control group treated with losartan; OB (n = 6): obese group; OBL (n = 6): obese group treated with losartan; * p<0.05 vs. C group; #p<0.05 vs. OB group; ANOVA and Tukey's test.

In regard to molecular responses, total protein expression of angiotensin (C 1.00±0.09; OB 0.66±0.11AU; p<0.05) and JNK (C 1.00±0.07; OB 0.55±0.06AU; p<0.05) were lower and ERK (C 1.00±0.13; OB 1.73±0.05AU; p<0.05) was higher in OB than C. As an independent factor, Losartan reduced angiotensin expression in both L groups (p<0.05; CL 0.62±0.04; OBL 0.56±0.10AU). With respect to MAPK, Losartan normalized obesity effects, as ERK (CL 1.07±0.07; OBL 1.23±0.20AU) and JNK (CL 0.74±0.13; OBL 0.87±0.17AU) expression values were similar (p<0.05) in CL and OBL groups (Figure 4).

**Figure 5:**
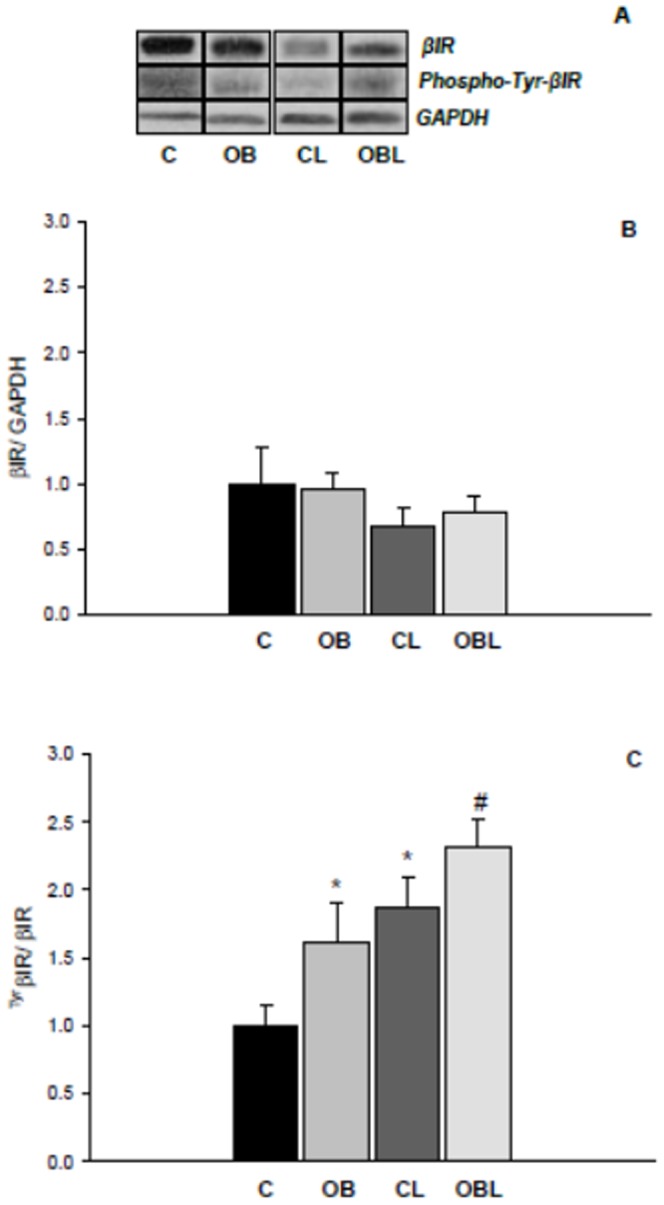
Protein levels of β-subunit of insulin receptor (βIR) in total and tyrosine phosphorylated forms in myocardial tissue; values presented as mean ± standard deviation. Total protein levels were normalized to GAPDH levels. (A) proteins bands for βIR, phosphor-Tyr-βIR and GAPDH; (B) βIR expression values; (C) phospho-Tyr-βIR expression values; C (n = 6): control group; CL (n = 6): control group treated with losartan; OB (n = 6): obese group; OBL (n = 6): obese group treated with losartan; * p<0.05 vs. C group; #p<0.05 vs. OB group; ANOVA and Tukey's test.

**Figure 6:**
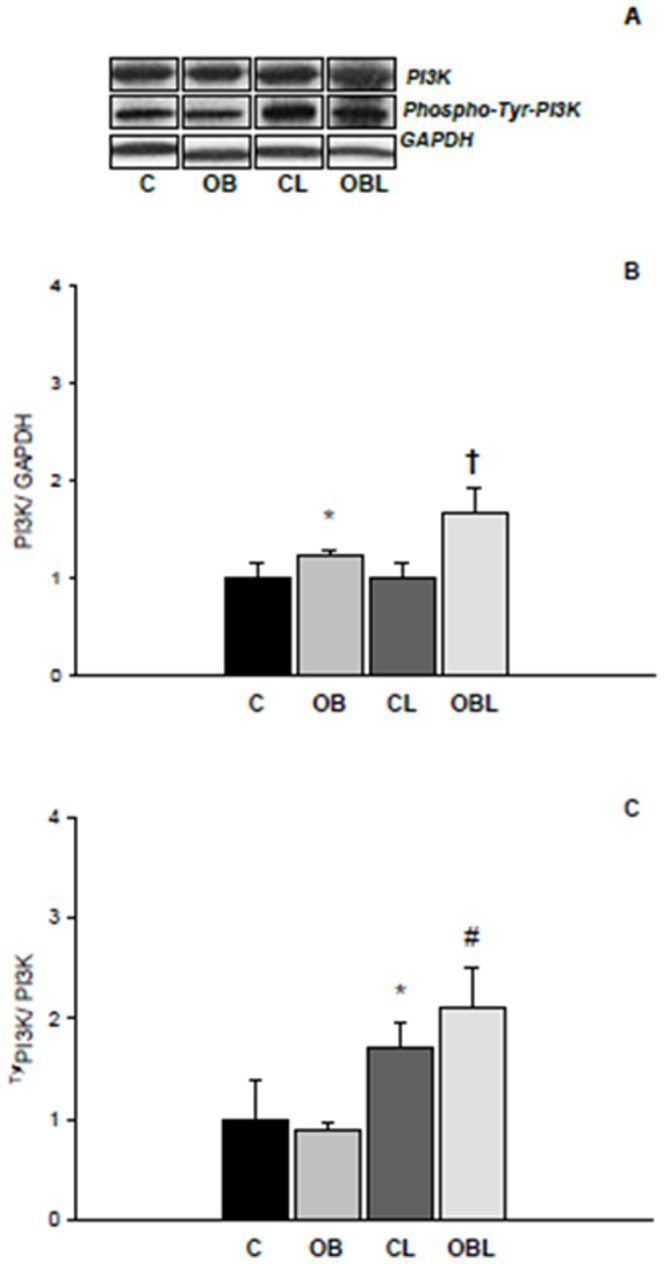
Protein levels of p85α subunit of PI 3-kinase (PI3K) in total and phosphorylated tyrosine forms in myocardial tissue; values presented as mean ± standard deviation. Total protein levels were normalized to GAPDH levels. (A) protein bands for PI3K, phosphor-Tyr-PI3K, and GAPDH; (B) PI3K expression values; (C) phospho-Tyr-PI3K expression values; C (n = 6): control group; CL (n = 6): control group treated with losartan; OB (n = 6): obese group; OBL (n = 6): obese group treated with losartan; * p<0.05 vs. C group; #p<0.05 vs. OB group; † p<0.05 vs. CL group; ANOVA and Tukey's test.

Also, although total Rβ expression was unchanged with obesity, OB exhibited higher Rβ expression in *Tyr*-phosphorylated form; importantly, Losartan increased *Tyr-phospho*-Rβ form expression in both L groups (C 1.00±0.15; OB 1.61±0.29; CL 1.87±0.23; OBL 2.32±0.20AU; Figure 5). Obesity was also associated with higher total PI3-kinase expression (C 1.00±0.15; OB 1.23±0.06; CL 1.01±0.14; OBL 1.68±0.25AU); however *Tyr-phospho*-PI3-kinase expression remained unchanged with obesity (C 1.00±0.39; OB 0.90±0.07; CL 1.71±0.26; OBL 2.11±0.39AU). Losartan independently increased total PI3-kinase expression in OBL and improved *Tyr-phospho*-PI3-kinase expression in both medicated groups (Figure 6).

## Discussion

Our initial hypothesis stated that obesity is associated with metabolic and cardiovascular disorders, including remodeling and insulin resistance in the heart. Our results confirmed the occurrence of several abnormalities including metabolic and endocrine disturbances as well as cardiovascular effects common to the present experimental model [4]–[8]. These effects were associated with MAPK activation and biochemical evidence of insulin resistance in the myocardium under the diet-induced obesity condition; importantly, a substantial part of these disorders were minimized with Losartan administration.

Obesity was induced by hypercaloric intervention with enhanced fatty acid content, including unsaturated and saturated lipids combined with sucrose overload, similar to a cafeteria diet [4]. Biometrically, both OB groups consisted of obese animals with higher body weight and adiposity values compared to their control counterparts. These effects were directly associated with improved feed efficiency in the OB groups although their calorie intake was lower (Table 1). Although this result is at variance with previous studies, the “hypophagia” observed with obesity may be due to the high serum leptin level (Table 1). Leptin, a hormone synthesized and secreted by adipose tissue, is mainly involved in the regulation of appetite and energy metabolism [38]. Particularly, the concept of selective leptin resistance might explain in part how hyperleptinemia could be accompanied by loss of appetite in the obese animals, despite body weight and adiposity levels in the OB groups. Actually, obesity is based on a disturbed energy balance, primarily through increased intake or decreased expenditure of energy [39].

Importantly, Losartan administration did not affect biometric and nutritional performance; comparatively, few studies [9], [40]–[43] have shown the impact of AT1R blockade interventions on biometric profile in rats with diet-induced obesity. Only Rosseli et al. [42] showed that losartan promoted a significant decrease in body weight and adiposity in a 20 week dietary protocol. However, similar to our study, other investigators have shown that AT1R blockade at 10 mg/kg/day [40], 20 mg/kg/day [41], [43], and 30 mg/kg/day [9] also did not affect nutritional behavior or biometric variables of rats submitted to 10 [41] or 16 week [9], [40], [43] protocols. In our study in particular, although absolute body weight and adiposity values were altered with AT1R blockade, calorie intake and feed efficiency between the 30^th^ and 35^th^ were reduced with medication. Possibly, the short intervention period (5 weeks) was not long enough to provoke more important modifications in nutritional and biometric profile, despite the substantial AT1R blockade dosage (30 mg/kg/day) used in this experiment.

Our results also showed that obesity, on its own, was associated with several metabolic and endocrine alterations, including dyslipidemia, hyperglycemia, insulin resistance, and hyperleptinaemia. These results and nutritional and biometric effects have been commonly seen in different experimental diet-induced obesity models [4]–[9], [40]. Insulin signaling disorders are associated with metabolic alterations in macronutrients and after hyperinsulinemia [10], [15]. Pharmacologically, losartan normalized the glycemia and insulin changes due to obesity, but OBL sustained superior HOMA values and impaired insulin sensitivity, as seen by ITT's AUC in relation to CL. These findings partially support the initial study hypothesis as ANG II configures a heterologous modulator of the insulin pathway [12], [14]. Therefore, other agents common to obesity, such as neuroendocrine, inflammatory, and dietary factors [17] could be responsible for these metabolic disturbances in the OBL group.

The occurrence of high serum cholesterol, triacylglycerol, and non-esterified fatty acid levels in the OB group can be explained by the chronic intake of lipids and sugar associated with renin-angiotensin system activation and insulin resistance in the obesity condition [15], [17]. Since ACE activity also increased in obese animals and AT1R antagonism was shown to regulate lipidemic balance in OBL, it is possible to conclude that angiotensin II plays an important role in the occurrence of dyslipidemia in these obese animals. Similar results have been documented in genetic obesity models submitted to AT1R blockade interventions [22]–[24]. Specifically, in Du Toit et al. [9], AT1R blockade resulted in glucose level normalization although it did not change dyslipidemia in rats with cafeteria diet-induced obesity. Differently, cholesterol and leptin levels increased with obesity, but were not affected by losartan administration. Clinical [19], [20] and experimental [44] studies have shown that cholesterol levels are generally determined by dietary concentration and are unaffected by AT1R blockade. Leptin is synthetized by adipose tissue and its concentration has been more correlated to the degree of body adiposity in experimental [45] and clinical [46] studies. ACE activity increased in the CL and decreased in OBL in relation to their control counterparts, supporting a significant interaction between obesity and losartan (p<0.05). The increased activity in CL could indicate an enzymatic feedback mechanism due to AT1 receptor blockade. However, losartan may be attenuating hepatic formation of angiotensinogen and/or rennin secretion in the kidneys of OBL animals which could reduce angiotensin I formation, and consequently ACE activity [47].

From the cardiovascular aspect, diet-induced obesity was associated with increased systolic blood pressure and some evidence of cardiac remodeling. Many studies have shown that dietary obesity is accompanied by vascular hyperactivity, an important mechanism in the installation of arterial hypertension [17]. Additionally, OB showed greater diastolic interventricular septal, left ventricular posterior diastolic wall, and relative thicknesses, as well as improved endocardium fraction shortening, ejection fraction, and posterior wall shortening velocity in relation to C (Table 2). Concerning contractile performance, systolic performance is related to factors such heart rate, contractility, preload, and afterload [48]. Although obesity did not have an impact on heart rate and LVEDd, increased wall thickness could be preserving or even decreasing cardiac preload; however, decreased preload would lead to reduced left ventricular ejection fraction [46], which was not found. Improved systolic function may therefore have resulted from modified afterload, but more probably from left ventricular hypertrophy. Afterload is a mechanical parameter directly influenced by ventricular pressure and diameter, and inversely related to wall thickness [1]. Persistently increased arterial pressure has been associated with greater afterload, parietal deformation, and cardiac hypertrophy [1], [48]. In this context, all the *in vivo* morphological and morphometric evidence are consistent with left ventricular concentric hypertrophy [1], [48]. Moreover, obese animals also showed higher collagen interstitial fraction levels and higher β-MyHC isoform expression than the C group. These effects have been associated with severe cardiac remodeling and could be indicative of diastolic dysfunction from a restrictive filling pattern [49]. However, with respect to ventricular performance, biometric condition did not alter diastolic function. A previous study revealed similar results after a 20 week experimental period [7].

Intriguingly, although β-MyHC composition did increase in obesity, left ventricular posterior wall shortening velocity was improved in the OB group. Generally, β-MyHC synthesis is associated with a reduction in myocardial contractile velocity and lower ATPase activity during the contractile cycle [50]–[52]. This dissociation between MyHC and functional performance results could be derived from factors such as variations in myocardial composition. In this context, previous studies [51], [52] have revealed that β-MyHC level is irregular in the heart and is more concentrated in the anterior wall next to papillary muscles. Instead, echocardiographic analysis allows global assessment of cardiac morphology and function without detecting regional differences in contractile performance [53]. These peculiarities can explain the differences between the results of systolic performance and MyHC distribution.

Associated to these alterations, obese animals presented lower angiotensin and MAPK-JNK expression coupled with high MAPK-ERK expression in the myocardium (Figure 3). Reduced angiotensin expression could be indicating increased peptide degradation of protein precursors in cardiac tissue during obesity. This event has been associated with greater ACE-2 activity derived from AT1 stimulation [54]. ACE2 is a new homolog of ACE that favors the degradation of Ang II to Ang (1–7) [54], which is not preferentially detectable with polyclonal antibody (sc-7419). This could therefore possibly support the reduction of intracellular Ang II levels; however this is only a supposition. Indeed, this process is a potential mechanism for extracellular conversion of the Ang II to Ang (1–7). Importantly, there was increased serum ACE activity in OB and CL groups. Generally, Ang (1–7) binds to Mas receptors and is considered a beneficial peptide of the RAS cascade in the cardiovascular system. ACE2 also cleaves Ang I to Ang (1–9), which is further converted by ACE into Ang (1–7). Acting on Mas receptors, Ang (1–7) might attenuate the actions of the ACE–Ang II–AT1R axis in cytosol [54]. Moreover, cardiac remodeling markers, such as myocardial hypertrophy and interstitial fibrosis, have been associated with elevated gene expression and increased ACE2 activity in the heart of nephrectomized [55] and infarcted rodents [56], and could be present in the OB group.

In relation to MAPK signaling, these proteins can be regulated by several and different molecular agents [18]. While ERK activation is more linked to growth factors and hormonal influence, JNK and p38K are particularly stimulated by mechanical deformation [10], [18]. Moreover, when one MAPK subtype is activated, inhibitory mechanisms related to phosphatase action have been shown to modulate the other MAPK subtypes [57]. Therefore, the respective increase in ERK accompanied by reduced JNK expression in our study may be supported by specific activation and inhibition mechanisms of these two MAPK family members. In this aspect, ERK activation could result from cross-talk mechanisms linked to hyperinsulinaemia and AT1R stimulation [12]–[14], [25] as postulated in the initial hypothesis. Growth and remodeling responses to insulin generally involve MAPK signaling pathways with *Shc* binding to the SH2 domain of Grb-2, which results in activation of the pre-associated GTP exchange factor Son of Sevenless (SOS) and GTP-binding protein *Ras*, which phosphorylate/activate MEK, and MAPK-ERK [12]. In support of this proposition, obesity was also related to greater expression of the phosphorylated form of the insulin receptor (Figure 4) and total PI3K (Figure 5), indicating that hyperinsulinaemia induced higher stimulation of the second messengers to insulin action. However, PI3K phosphorylation was not modified with these alterations, suggesting the occurrence of insulin resistance in the heart. Carvalheira et al. [25] documented similar responses the myocardial tissue of Zucker obese rats.

A large number of endocrine, inflammatory, neural, and cell-intrinsic pathways have been shown to be dysregulated in obesity. Although it is possible that one of these factors plays a dominant role, many are interdependent, and it is likely that their dynamic interplay underlies the pathophysiology of insulin resistance [17]. In our study, AT1R blockade administration promoted normalization of the metabolic and cardiovascular disturbances, including cardiac remodeling and insulin resistance, arising from hypercaloric diet-induced obesity. These findings support the occurrence of cross-talk between insulin and AT1R mechanisms from metabolic and cardiovascular perspectives and confirm our initial hypothesis. Cross-talk from heterologous receptor signaling pathways, such as AT1R, exert enhancing effects on these growth/remodeling signaling pathways whilst interfering with the metabolic signaling pathway [14], [15].

AT1R activation induces several pleiotropic effects from angiotensin II, such as peripheral vasoconstriction, aldosterone secretion, sodium reabsorption, and arterial hypertension [12]. Therefore, AT1R blockade affects peripheral resistance, increasing blood irrigation in skeletal muscle and improving glycemic metabolism [20], [21]. Similar beneficial responses have been described in rodents submitted to fructose overload [45] and cafeteria diet [9], which support the normalization of arterial pressure and glycemic metabolism seen in our OBL group. Moreover, acute or chronic reductions in systolic blood pressure have been associated with adaptive mechanisms destined to control arterial pressure [58]; in this context, heart rate elevation with the Losartan administration, *per se*, could be associated with sympathetic activation conjugated to parasympathetic inhibition through arterial baroreceptors, to compensate for decreased peripheral resistance. As a consequence, E/A, IVRT, and R-R interval were reduced with losartan in both medicated groups. These responses may be related to increased sarcolemmal influx of Ca^2+^ into cytosol and an increase in the rate of adenosine triphosphate (ATP) hydrolysis, with a positive inotropic effect on myocardial performance [48] designed to compensate for AT1R blockade with losartan.

With respect to contractile performance, since systolic function was normalized in the OBL group, it is acceptable that AT1R blockade attenuated the myocardial inotropic effect derived from the endocrine role of adipose tissue, with a consequent reduction in myofibril and sarcomere recruitment; actually, ventricular hypertrophy was controlled in OBL, as supported by macro and microscopic evidence. These morphological and functional findings have been directly associated with MAPK-ERK activation maintained by AT1R stimulation. Similarly, collagen interstitial fraction was reduced with losartan administration even though there are differences between CL and OBL due to the influence of obesity. Several studies have shown that AT1R activation is associated with interstitial fibrosis and that collagen accumulation is directly associated with ventricular hypertrophy [10]. These results support the fact that cardiac remodeling in our experimental model is strongly associated with angiotensin-aldosterone-rennin system activation, although MyHC effects were not affected by losartan administration. In this specific case, other agents, including hyperleptinaemia [17], dietary sucrose, and/or lipids could potentially be related to β-MyHC synthesis in the obese animals.

In conclusion, AT1R blockade attenuated several metabolic and cardiovascular disturbances, including dyslipidemia, glycemic alterations and insulin resistance, and phenotypical responses in myocardial tissue from obese animals. Cardiovascularly, losartan normalized arterial pressure disturbances and molecular mechanisms for manifestation of remodeling and insulin resistance in the heart.
